# Augment Reality-Based Teaching Practice

**DOI:** 10.1007/s43683-020-00040-x

**Published:** 2020-11-11

**Authors:** You Wu, Mingzhen Zhang, Xiaosi Li, Yu Gan, Chao Zhao

**Affiliations:** 1 Department of Chemical and Biological Engineering, The University of Alabama, P. O. Box870203, Tuscaloosa, AL 35401 USA; 2 Computational Structural Biology Section, Basic Science Program, Frederick National Laboratory for Cancer Research, Frederick, MD 21702 USA; 3 Department of Electrical and Computer Engineering, The University of Alabama, P. O. Box870286, Tuscaloosa, AL 35401 USA

## Challenges

The COVID-19 pandemic has shut down society in an unprecedented way. The past Spring 2020 semester has witnessed a rapid transition from in-person to online teaching. For teachers and students, such transition has been progressing with challenges, especially for courses that usually require laboratory settings. Here, we describe the challenges that we encountered during such transition and discuss a virtual laboratory setting based on augmented reality (AR) to improve online learning.

The challenges concentrate on *hands-on skill learning*, *knowledge gain*, and *social interaction*.

### Hands-On Skill Learning

Lack of access to laboratory facilities, a key loss after transition to online teaching, imposes the first challenge particularly in courses requiring a wet laboratory. With a short responding time to COVID-19 and a rapid transition to online teaching, we were not able to secure a solution that would enable the students to visualize or mimic the process of data acquisition. Instead, we were left with the option that distributes previously acquired data with modifications to students for laboratory reports purpose. Consequently, students have to skip experimental setup, which may prevent them from understanding the mechanisms of how the instruments deliver the designated measures. Also, students are fed with data to complete laboratory reports, rather than engaging in acquiring data independently or collaboratively. Students essentially have the minimal, if not zero, opportunity to strengthen their on-the-ground observational skills.

### Knowledge Gain

A physical classroom setting enables vivid instruction of concepts/ideas that span into a three-dimentional (3D) space with geometry, morphology, and texture features. The instructor could utilize a model or demo to explain during lectures. In an online class, unfortunately, it is converted into a plain, verbal delivery of messages and additional teaching strategy will need to be implanted to stimulate students’ conceptual digestion. Moreover, it has been shown that distraction becomes easier in audio settings without actual eye contact,^1^ a fact that could cause delays for students to receive the messages. Also, the online pattern provides minimal physical space change (e.g. classroom from one teaching building to another) but constrains the students in their chosen space and it could worsen tediousness, especially when multiple courses are scheduled in a row with minimal breakups.

### Social Interaction

Students show varying degrees of decline in learning satisfaction due to the limited teaching-learning interactions in online classes.^2^ Two realities impose negative impacts on quality learning. First, instructions from the lecturer and instantaneous responses from students are no longer in an immediate two-way fashion. Communications in online classes are largely determined by network speed and real-time performance of the platform (e.g. Zoom software). The lecturers could essentially be instructing in a one-way silent mode as responses from students are usually delayed or even lacking. In addition, the use of a camera in an online classroom is generally optional but not required. Lacking facial expressions in communication could potentially diminish the message being delivered.^3^ In such scenarios, both lecturers and students experience less satisfaction. Second, team-work training is essentially not applicable and students reportedly feel that learning in an isolated community does not promote understanding.^4^,^5^ This reality is deeply challenged in collaborative laboratory projects. Even though the availability of online group discussions could serve as a compensating mechanism, the lack of co-operation on the same experimental subject is not replenishable and each student assigned in a group project will have to largely work on his or her own part with minimal or no interaction with partners.

## Novel Initiative

**Figure 1:**
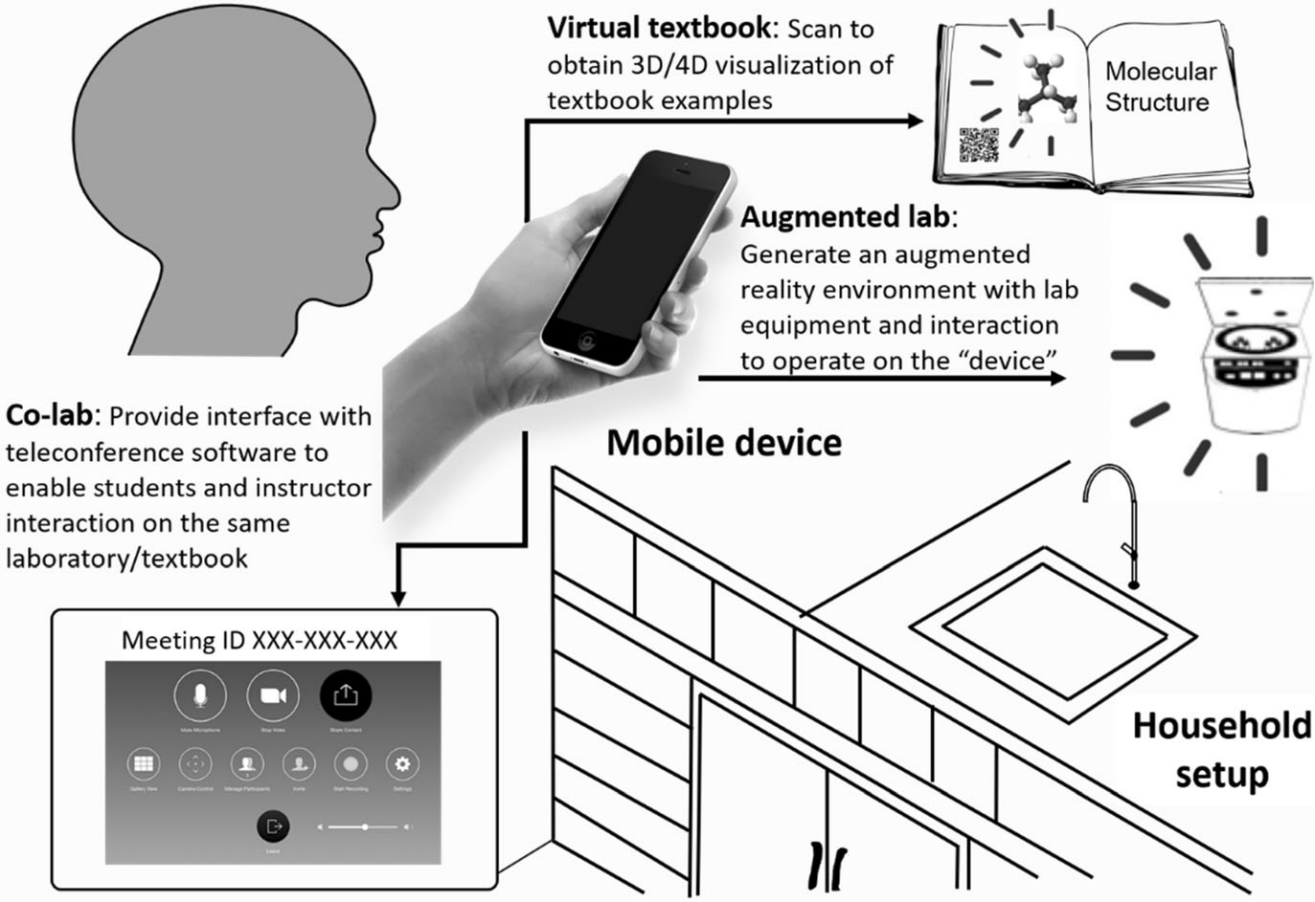
System overview. Virtual content (virtual lab device or 3D books content) is added to the real world (table). A hardware device (smartphone or tablet) is used to make the content visible for the user. The software also includes interface to teleconference software, such as Zoom or Skype.

To address the above challenges, we propose a novel online teaching solution based on AR technology. The proposed system is shown in Fig. 1. The whole system requires only hardware of mobile device (smartphone or tablet). We expect to implement an AR-based platform with three modules: *augmented lab*, *virtual textbood*, and *co-lab*. We foresee such immersive technologies could provide a “real” lecturing and laboratory environment.

*Novelty*: First, within immersive technology, we choose AR over virtual reality (VR) due to AR’s advantages in cost, accessibility, and user engagement. Any student could simply download the application from his or her mobile device and interact with the software as playing an AR game, such as Pokémon GO. If it is implemented in VR, a virtual setting that suits the needs of most students will need to be designed, tested, and implemented, a process that will elongate our study and increase cost; additionally, an extra headset, usually pricing at $400~$600, is required thus not widely feasible to implement at home during COVID-19. Moreover, AR could enhance the user engagement and interaction in a daily house-setting, minimizing of impact of environment changes on students’ learning. Second, our design explores a new realm of AR application for educational purpose. Existing AR technology dominantly focuses on medical education^6^ with a demonstration of static visualization anatomy. In contrast, our design focuses on the understanding of engineering laboratory instruments and textbook knowledge with an addition of dynamic practical functionality of social attributes.

### Augmented Lab

**Figure 2:**
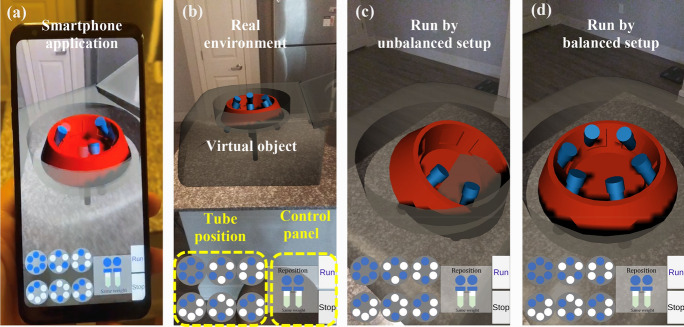
Screenshot of AR demo. (**a**) Smartphone application; (**b**) the layout of application; (**c**) demo of a virtual experiment run by unbalanced setup; (**d**) demo of a virtual experiment run by balanced setup

We will develop an AR module named augmented lab to address the concerns for lacking access to laboratory settings that train hands-on skills. We develop the laboratory by following publicly available resources.^7^ Benefiting from its open-sources feature, we choose to use the Android system as the platform to develop the AR module, we choose Unity integrated development environment (IDE) as a gaming engine to develop a smartphone application for users. To enhance the visualization, we make augmented models in Blender and then export them to Unity. We present a preliminary example of this prototype with an AR-based centrifuge (Fig. 2, Supplementary Video). This virtual centrifuge will be added to real-world household setup (e.g. table). It will allow a set number of students to operate on each student’s computer end. In such a scenario, each student will be able to balance his or her microtubes, optimize the centrifuge settings, and run the machine per their experimental protocol (Fig. 2b). In the case of an unbalanced setting (Fig. 2c), the tubes will not be properly centrifuged. More instruments will be implemented within our computational capability to maximize a virtual experimental setup and test.

### Virtual Textbook

We are currently ongoing the implementation of a virtual textbook to provide better virtual visualization for online teaching. We propose to establish virtual textbooks that are scannable via Quick Response (QR) codes such that students have access to a 3D demo of the complex concepts such as molecular structure of proteins, polymers, and nucleic acids, etc. We will enhance a scalable visualization for histology such that students can freely zoom-in and zoom-out to visualize pathological microstructure at different scales while reading books or attending online classes. The scalable and rotatable visualization function allows students to visualize the structure with different proportions and angles. Such demo could help deepen students’ understanding of the textbook and raise students’ curiosity in learning new knowledge and building their habits of thinking in 3D or multi-scale visualizations.

### Co-lab

We will also implement social interaction features to the online teaching software. We will provide an interface with teleconference software to sync AR visualization with online group discussions (one-to-many) and virtual office hours (one-to-one). Online group discussions will be added to each course to ensure high-quality communication. For projects that require cooperation, the project leader will be encouraged to organize an online coffee club to enrich the discussion. The virtual office hour will have greater flexibility without specifing a fixed time and location. Instead, it could potentially be applied in the format of more frequent email exchanges with instructor, brief but efficient Zoom meetings with the instructor or teaching assistant, and more social media communication (if applicable).

## Reflection

We practiced in a wet laboratory course in our department, distributing students with previously acquired data to produce laboratory reports, a fashion of Behaviorism learning.^8^ This leads to reports that lack an in-depth description of experimental protocols and procedure discussions. There was only a minimal training for students to strengthen their observational skills and detect nuances between similar experimental procedures. We are thus inspired to develop a virtual co-lab to improve the learning satisfaction.

**Figure 3:**
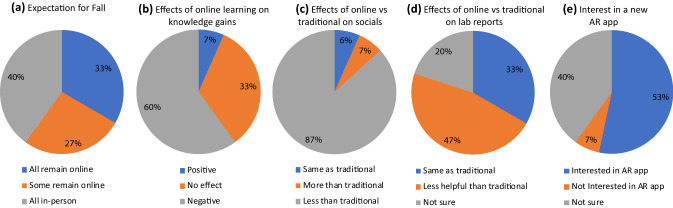
Quantifications of survey returns.

We performed a survey on online learning and expectation for the coming semesters. This survey has been given exempt approval by the institutional review board (IRB) committee for the reason that this survey serves as an educational investigation to compare effectiveness of instructional techniques. The inclusion criteria for data analysis are students who participated in an online class in the Spring 2020 and attend the Fall 2020 semester. We analyze and summarize the survey (n=15) as below in Fig. 3.

The three challenges are well supported by survey results. In terms of hands-on skill learning and submitting laboratory reports, only 33% of students rate online classes as equally helpful as traditional classes while 67% rate online less helpful. In terms of knowledge gains, 93% of students do not rate online classes as positive. Closely, 87% of students reported that social interaction is heavily impaired and needs improvement. All students express the need for more virtual office hours and online group discussions if online teaching will continue. Taken these practices into consideration, 67% of students express expectations for in-person instructions for at least a part of Fall semester. In sum, 40% of students rate their Spring online learning as positive, albeit the abovementioned challenges were explicitly presented. Likely contributing factors to positive experience may include more flexibility in assignment submission, less commute for attending class, and the availability of audio content for off-campus review.

We will practice constructivism experiential learning in Fall 2020 semester to promote students learning in an active, contextualized process^9^. We also observed that students who had AR-based gaming experience expressed interests in having AR-based virtual laboratory settings as a part of new semester, and that 30% of those who had no AR experience showed some interest. We attribute this yes-and-yes-no-maybe-yes phenomenon to that AR experience exposes students with direct visualization and sensory satisfaction, thus anyone may need to have AR experiences to become interested in AR. Hence, we will integrate AR into laboratory settings and practice constructivism learning.

We set two minimal learning objectives to test whether or not AR-based laboratory enhances learning. First, without operating on actual instruments, AR-lab will deepen students’ understanding in setting up and calibrating instruments correctly. For example, students need to virtually balance the centrifuge and load tubes. Second, students become capable of identifying virtual experimental readings that cannot be generated due to incorrect operations. Upon the implementation of new AR platform, we will assess the success of our initiatives. The attainment of student outcomes of upcoming semesters will be evaluated, scored, and compared with the Spring 2020 results. Specifically, to assess the success of three AR-based teaching modules, students’ laboratory skills, communication skills, shifts in students’ attitudes towards online class, the shift in behaviorism/constructivism, and teamwork skills will be evaluated. The detailed performance criteria will include an experimental plan, data collection, data analysis, data presentation, and conclusion.

By comparing the student outcomes between with and without AR, we will employ an evidence-based approach to improve the current AR teaching strategy. Specifically, we will implement a better AR demo or add new features to the existing demo. In the future, after the COVID-19 pandemic is no longer a concern, this practice could still be used as an alternative for those who are not able to make their presence in laboratory.
